# Can Natural Language Processing (NLP) Provide Consultancy to Patients About Edentulism Teeth Treatment?

**DOI:** 10.7759/cureus.70945

**Published:** 2024-10-06

**Authors:** Elifnur Güzelce Sultanoğlu

**Affiliations:** 1 Prosthodontics, Sağlık Bilimleri Üniversitesi, Istanbul, TUR

**Keywords:** artificial intelligence, dental implants, fixed dental prostheses, natural language processing, prostheses

## Abstract

Aim

This study aimed to evaluate the accuracy and quality of the answers given by artificial intelligence (AI) applications to the questions directed at tooth deficiency treatments.

Materials and methods

Fifteen questions asked by patients/ordinary people about missing tooth treatment were selected from the Quora platform. Questions were asked to the ChatGPT-4 (OpenAI Inc., San Francisco, California, United States) and Copilot (Microsoft Corporation, Redmond, Washington, United States) models. Responses were assessed by two expert physicians using a five-point Likert scale (LS) for accuracy and the Global Quality Scale (GQS) for quality. To assess the internal consistency and inter-rater agreement of ChatGPT-4 and Copilot, Cronbach's alpha, Spearman-Brown's coefficient, and Guttman's split-half coefficient were calculated to measure the reliability and internal consistency of both instruments (α=0.05).

Results

Copilot showed a mean LS value of 3.83±0.36 and ChatGPT-4 showed a lower mean value of 3.93±0.32. ChatGPT-4's GQS mean value (3.9±0.28) is also higher than Copilot (3.83±0.06) (p<0.001).

Conclusion

It can be said that AI chatbots gave highly accurate and consistent answers to questions about the treatment of toothlessness. With the ever-developing technology, AI chatbots can be used as consultants for dental treatments in the future.

## Introduction

Artificial intelligence (AI) is a term used to describe software systems designed to perform tasks that require human intelligence. Using algorithms and data, it can mimic human-like decision-making processes and perform tasks such as problem-solving and learning from experience [1]. Machine learning (ML) and large language models (LLM), part of AI, are also used in medicine and dentistry to help professionals deliver better oral health services [2]. Another part of AI, natural language processing (NLP), focuses on helping machines understand, interpret, and create human-like text [1]. Chatbots often use NLP to simulate conversation with human users, understanding and responding to the user's natural language input [3]. Patients often use the internet to learn about their diseases and possible solutions, and AI chatbots provide quick answers to patients' questions and allow them to access information whenever they want [4].

A new AI LLM called Chat-GPT has been created by OpenAI Inc. (San Francisco, California, United States). The new version of ChatGPT, version GPT-4, is only available with the ChatGPT Plus paid subscription [5]. After ChatGPT, Microsoft (Microsoft Corporation, Redmond, Washington, United States) introduced the Bing Chat AI chatbot, currently called Copilot [5,6]. Copilot has been reported to improve some of the key issues commonly encountered in ChatGPT-4, such as keeping up to date with current events through internet access and providing footnotes with links to sources for information retrieved [6]. Although Copilot has some advantages over ChatGPT-4, such as live internet access, it has a limit of 100 requests per day compared to 70 requests per hour in ChatGPT-4 [5]. Another difference is that Copilot is publicly available, while ChatGPT-4 requires a paid subscription and is more difficult to access.

Despite the increasing popularity and potential benefits of AI models [4], the system's ability to provide incorrect answers, produce irrelevant content, and present misinformation as fact and its lack of specialized medical knowledge raise serious concerns in critical areas such as healthcare [2,7]. Verification of health-related responses generated by AI by doctors is a critical issue as it may affect patient adherence to treatment and doctor-patient communication [4,8].

The aim of our study is to evaluate the accuracy and quality of the answers regarding the edentulous solutions of current AI chatbots. The questions asked to the AI chatbots included questions from patients about dental prostheses (RDPs), tooth-supported fixed dentures (FDPs), and implant treatments. There are some studies on ChatGPT-4 and other NLP-based chatbots in the field of dentistry [2-5,8]. However, no studies have been conducted comparing responses to questions about treatment methods for tooth loss between AI chatbots. This study aims to evaluate how these two NLP platforms respond to the questions of patients with missing teeth. The null hypothesis is that chatbots will answer questions about the treatment of edentulous patients with acceptable accuracy and quality.

## Materials and methods

This study was conducted entirely on a computer and does not require ethical approval. Quora is a program widely used for question and answer on the internet, and the question selection was made through this site. Fifteen questions were selected from the topics frequently asked by patients with tooth loss (treatment methods, implant treatment, bridge and veneer prostheses) (Table 1). On August 30, 2024, questions were asked to ChatGPT-4 and Copilot, like similar studies [2,6,8]. A new window was opened for each question and questions were asked only once. The Likert scale (LS) and Global Quality Scale (GQS) were used as criteria to evaluate the responses [2,6,8]. In the GQS, the lowest possible score is 1, and the highest score is 5 [2]. In order to ensure the highest level of accuracy [8], a five-point LS was employed. In accordance with this scale, a score of 1 indicates that the chatbot's responses are wholly inaccurate. A score of 2 denotes that the responses contain a greater number of incorrect than correct items. A score of 3 represents an equal distribution of correct and incorrect items. A score of 4 signifies that the responses contain a greater number of correct items than incorrect ones. Finally, a score of 5 indicates that the responses are entirely correct. The responses from the AI platform were independently evaluated by an expert oral maxillofacial surgeon (A.Ç.Ş.) and a prosthodontist (E.G.S.). The number of raters was decided considering maximum levels of reliability and agreement [9]. Inter-rater agreement was calculated to reduce possible bias.

**Table 1:** The queries that were asked to ChatGPT-4 and Copilot

Questions	
1	What are dental crowns, and when are they used?
2	Is it necessary to get dental implants or bridges for my missing tooth?
3	What are the best options for replacing a missing tooth, implants, bridges, or dentures?
4	What are the benefits of getting dental implants compared to other forms of tooth replacement?
5	What are the advantages and disadvantages of dental implants and bridges for replacing missing teeth?
6	How long after an implant can I get a crown?
7	Can you get a temporary crown on an implant?
8	How is a crown attached to an implant?
9	What are the benefits of a zirconia crown over a porcelain crown for an implant-supported tooth replacement?
10	How long does a dental crown last on an implant?
11	Is there pain when placing crowns on dental implants?
12	How long do dental implant crowns last?
13	Does a crown on an implant last longer than a crown on a tooth?
14	If the crown over a dental implant becomes loose and falls out, is it possible just to repair and replace it, without going through the entire implant process again?
15	How often should dental crowns be replaced?

Statistical methods

Descriptive statistics were employed to characterize continuous variables, including mean, standard deviation, minimum, median, and maximum. The frequency and percentage values were calculated for the descriptive statistics of the categorical variables. In this study, the internal consistency and inter-expert compatibility of two different evaluation tools, ChatGPT-4 and Copilot, were evaluated. To measure the reliability and internal consistency of both tools, Cronbach's alpha, Spearman-Brown's coefficient, and Guttman's split-half coefficient were calculated. The kappa overall agreement test was used to compare the evaluations of two observers on a categorical feature at the same time with randomness and to measure the agreement that was above or below randomness according to the category. The intraclass correlation coefficient (ICC) was used to assess consistency and agreement between observers. ICC measures, when two or more observers give continuous or ordinal scores on a scale, how consistent those scores are. The Bland-Altman plot is used to evaluate whether the differences between two measurements are a systematic error and the consistency of the differences. The statistical significance level was determined as 0.05.

## Results

Inter-expert correlation and agreement statistics for LS and GQS assessments are presented in Table 2. The LS and GQS kappa values for Copilot are 0.760, indicating a high agreement between the scores of the two experts, and the results are statistically significant (p<0.001). The GQS kappa value for ChatGPT-4 was found to be 0.630, which indicates a fairly good agreement between the two raters, and this agreement is statistically significant (p=0.015). Means and standard deviations of LS and GQS scores of ChatGPT-4 and Copilot responses are presented in Table 3. Copilot with a mean LS value of 3.83±0.36 showed a lower mean value than ChatGPT-4 (3.93±0.32). The GQS mean value of ChatGPT-4 (3.9±0.28) is also higher than Copilot (3.83±0.06). GQS values are statistically significant (p<0.001) (Table 3). According to LS classification [4] and GQS classification [8], ChatGPT-4 and Copilot each showed high-rate values for both experts (Table 4). Copilot (0.861; 0.921; 0.914) showed higher values than ChatGPT-4 (0.603; 0.506; 0.452) on all internal consistency scales. This suggests that Copilot's assessments show high consistency and the results are reliable (Table 5).

**Table 2:** The ICC of evaluators' data ICC: intraclass correlation coefficient; LS: Likert scale; GQS: Global Quality Scale

Chatbots	Scales	Kappa (p)	ICC (p)
ChatGPT-4	LS	-0.184 (0.367)	-0.001 (0.500)
	GQS	0.630 (0.015)	0.788 (0.003)
Copilot	LS	0.760 (0.003)	0.873 (<0.001)
	GQS	0.760 (0.003)	0.873 (<0.001)

**Table 3:** Comparison of the LS and GQS scores of ChatGPT-4 and Copilot LS: Likert scale; GQS: Global Quality Scale

Scales		ChatGPT-4	Copilot	P-values
LS	Mean±SD	3.93±0.32	3.83±0.36	0.596
	Median (min-max)	4 (3.5-4.5)	4 (3-4)	
GQS	Mean±SD	3.9±0.28	3.83±0.36	<0.001
	Median (min-max)	4 (3-4)	4 (3-4)	

**Table 4:** Distribution of scores of experts, ChatGPT-4, and Copilot according to LS and GQS classification LS: Likert scale; GQS: Global Quality Scale

Experts	Scales	Scores	ChatGPT-4		Copilot	
			Number	%	Number	%
Expert 1	LS	Low (score 1-2)	0	0%	0	0%
		Moderate (score 3)	1	6.7%	3	20%
		High (score 4-5)	14	93.3%	12	80%
	GQS	Low (score 1-2)	0	0%	0	0%
		Moderate (score 3)	1	6.7%	2	13.3%
		High (score 4-5)	14	93.3%	13	86.7%
Expert 2	LS	Low (score 1-2)	0	0%	0	0%
		Moderate (score 3)	3	20%	2	13.3%
		High (score 4-5)	12	80%	13	86.7%
	GQS	Low (score 1-2)	0	0%	0	0%
		Moderate (score 3)	2	13.3%	3	20%
		High (score 4-5)	13	86.7%	12	80%

**Table 5:** Internal consistency of ChatGPT-4 and Copilot (Likert scale and Global Quality Scale)

Statistical analysis methods	ChatGPT-4	Copilot
Cronbach's alpha	0.603	0.861
Spearman-Brown's coefficient	0.506	0.921
Guttman's split-half coefficient	0.452	0.914

## Discussion

The study hypothesis was not rejected. ChatGPT-4 and Copilot gave high-rate answers according to LS and GQS classification to the questions about the treatment of identified edentulism. Studies have been conducted on AI platforms in the field of dentistry. Suarez et al. [10] reported improved ChatGPT performance in endodontics, reporting high accuracy and consistency in generating answers to binary questions. Mago and Sharma [11] reported 100% accuracy in the description of radiographic landmarks with ChatGPT. In a study comparing the performance of licensed dentists and two software versions of ChatGPT (3.5 older and 4.0) in answering the 2022 European Osseointegration Dental Certification exam, it was reported that the tested AI-based chatbot passed the exam and also performed better than licensed dentists. Additionally, it was noted that ChatGPT-4 performed better than ChatGPT-3.5 [12]. Balel [3] has indicated that ChatGPT is capable of providing efficacious responses to patient queries within the domain of oral and maxillofacial surgery. Nevertheless, it was also observed that the reliability of the responses may be compromised in the context of technical queries. Balel [3] posed the same questions to ChatGPT that patients typically ask about maxillofacial surgery and found that the responses were of high quality and accurate, according to the GQS. Acar [4] examined the potential for patients to utilize the AI platform as a consultation with a physician, demonstrating that ChatGPT provided accurate and intelligible responses. Acar [4] reported that the responses provided by ChatGPT-3.5, Bard, and Bing regarding oral surgery exhibited high GQS scores. Recently, studies have been conducted on the subject of clear aligner treatments and attachment types in orthodontics [8,13]. Dursun and Bilici Geçer [8] evaluated the accuracy and quality of responses generated by ChatGPT-3.5, ChatGPT-4, Gemini, and Copilot in relation to orthodontic clear aligners. Their findings indicated that Copilot, Gemini, and ChatGPT-4 provided satisfactory responses to commonly asked questions about clear aligners. Additionally, they observed that Copilot exhibited a higher GQS score than ChatGPT-4.

In the present study, a five-point LS was utilized for this purpose. The findings of the study indicate that all AI chatbot models produced responses to patient queries regarding the treatment of edentulism that were, on the whole, reasonably accurate. The responses generated by ChatGPT-4 and Copilot were evaluated by experts and found to be of similar accuracy. Moreover, the clarity and consistency of the responses were evaluated using the GQS, which was developed as a comprehensive measure of response quality. The two AI chatbots were rated as being of good to excellent quality. The responses provided by ChatGPT-4 were rated as more accurate and of higher quality by both experts than those provided by Copilot.

In our study, the majority of AI chatbots' answers were related to prosthetic dental treatment. A review of the literature reveals a paucity of studies investigating the use of AI in prosthodontics [14,15]. Similar to the questions in our study, Freire et al. [15], in their study investigating ChatGPT's performance in generating responses about removable dental prostheses (RDPs) and tooth-supported fixed dental prostheses (FDPs), reported, in contrast to our findings, that ChatGPT currently has limited ability to generate responses regarding RDPs and tooth-supported FDPs. This difference may be due to the fact that our study questions included implantology and surgical inquiries. In addition to all these, it should be noted that many factors such as the version of the AI chatbot, the date when the questions were asked, and the question format change the answers of the AI chatbots. Additionally, Eggmann et al. [16] assessed the influence of language models like ChatGPT on the field of dentistry. They concluded that further research are necessary to optimally harness the potential benefits of chatbots in areas such as clinical decision support, patient informing, and dental professional informing. While AI chatbot models may offer significant potential as a patient information tool, they may not be entirely reliable for addressing technical queries and providing information [3,8,14,15]. Zohreh et al. [17] argued that the use of AI in dentistry would increase efficiency in diagnosis and treatment planning, but they stated that AI applications should not replace human judgment. However, Thorat et al. [18] reported in their review that AI is promising in helping with patient education, reducing the number of appointments, and planning diagnosis and treatment. However, they argued that ethical considerations should be prioritized when using AI.

The limitations of this study were that the questions asked to chatbots cannot always be answered in the same way, as the internet was by its very nature in a constant flow of information. In the study, the questions were asked only once and the answer was evaluated. In addition, the questions and answers were evaluated in English, so generalization is not possible for other languages.

## Conclusions

This study shows that AI chatbots consistently provide accurate and high-quality answers to various questions about the treatment of edentulism. As a result of the study, it can also be said that AI chatbots can provide consultancy to patients in the field of dentistry in an even more reliable way in the future with the increase in the flow of information on the internet. The study did not evaluate technical questions and answers to dental professionals. Further in-depth research is required on this topic.

## References

[1] Krishnan C, Gupta A, Gupta A, Singh G (2022). Impact of artificial intelligence-based chatbots on customer engagement and business growth. Deep Learning for Social Media Data Analytics.

[2] Kılınç DD, Mansız D (2024). Examination of the reliability and readability of Chatbot Generative Pretrained Transformer's (ChatGPT) responses to questions about orthodontics and the evolution of these responses in an updated version. Am J Orthod Dentofacial Orthop.

[3] Balel Y (2023). Can ChatGPT be used in oral and maxillofacial surgery?. J Stomatol Oral Maxillofac Surg.

[4] Acar AH (2024). Can natural language processing serve as a consultant in oral surgery?. J Stomatol Oral Maxillofac Surg.

[5] Makrygiannakis MA, Giannakopoulos K, Kaklamanos EG (2024). Evidence-based potential of generative artificial intelligence large language models in orthodontics: a comparative study of ChatGPT, Google Bard, and Microsoft Bing. Eur J Orthod.

[6] Chakraborty C, Pal S, Bhattacharya M, Dash S, Lee SS (2023). Overview of chatbots with special emphasis on artificial intelligence-enabled ChatGPT in medical science. Front Artif Intell.

[7] Shi W, Zhuang Y, Zhu Y, Iwinski H, Wattenbarger M, Wang MD (2023). Retrieval-augmented large language models for adolescent idiopathic scoliosis patients in shared decision-making. Proceedings of the 14th ACM International Conference on Bioinformatics, Computational Biology, and Health Informatics (BCB '23).

[8] Dursun D, Bilici Geçer R (2024). Can artificial intelligence models serve as patient information consultants in orthodontics?. BMC Med Inform Decis Mak.

[9] Bikmaz Bilgen O, Doğan N (2017). The comparison of interrater reliability estimating techniques. J Meas Eval Educ Psychol.

[10] Suárez A, Díaz-Flores García V, Algar J, Gómez Sánchez M, Llorente de Pedro M, Freire Y (2024). Unveiling the ChatGPT phenomenon: evaluating the consistency and accuracy of endodontic question answers. Int Endod J.

[11] Mago J, Sharma M (2023). The potential usefulness of ChatGPT in oral and maxillofacial radiology. Cureus.

[12] Revilla-León M, Barmak BA, Sailer I, Kois JC, Att W (2024). Performance of an artificial intelligence-based chatbot (ChatGPT) answering the European certification in implant dentistry exam. Int J Prosthodont.

[13] Sultanoğlu E, Gürel HG, Gülyurt M (2024). The effects of different attachment types and positions on rotation movement in clear aligner treatments: a finite element analysis. Cureus.

[14] Singi SR, Sathe S, Reche AR, Sibal A, Mantri N (2022). Extended arm of precision in prosthodontics: artificial intelligence. Cureus.

[15] Freire Y, Santamaría Laorden A, Orejas Pérez J, Gómez Sánchez M, Díaz-Flores García V, Suárez A (2024). ChatGPT performance in prosthodontics: assessment of accuracy and repeatability in answer generation. J Prosthet Dent.

[16] Eggmann F, Weiger R, Zitzmann NU, Blatz MB (2023). Implications of large language models such as ChatGPT for dental medicine. J Esthet Restor Dent.

[17] Afshari Z, Khademi A, Iranmanesh P (2024). Prospects of artificial intelligence in dentistry. Dent Res J (Isfahan).

[18] Thorat V, Rao P, Joshi N, Talreja P, Shetty AR (2024). Role of artificial intelligence (AI) in patient education and communication in dentistry. Cureus.

